# Electrical Discharge in a Cavitating Liquid under an Ultrasound Field

**DOI:** 10.1021/acs.jpclett.3c02778

**Published:** 2023-11-30

**Authors:** T. Karabassov, A. S. Vasenko, V. M. Bayazitov, A. A. Golubov, I. S. Fedulov, A. V. Abramova

**Affiliations:** † HSE University, 101000 Moscow, Russia; ‡ Donostia International Physics Center (DIPC), 20018 San Sebastián/Donostia, Basque Country, Spain; § N.S. Kurnakov Institute of General and Inorganic Chemistry, Russian Academy of Sciences, 119991 Moscow, Russia; ∥ Faculty of Science and Technology and MESA+ Institute of Nanotechnology, University of Twente, 7500AE Enschede, The Netherlands

## Abstract

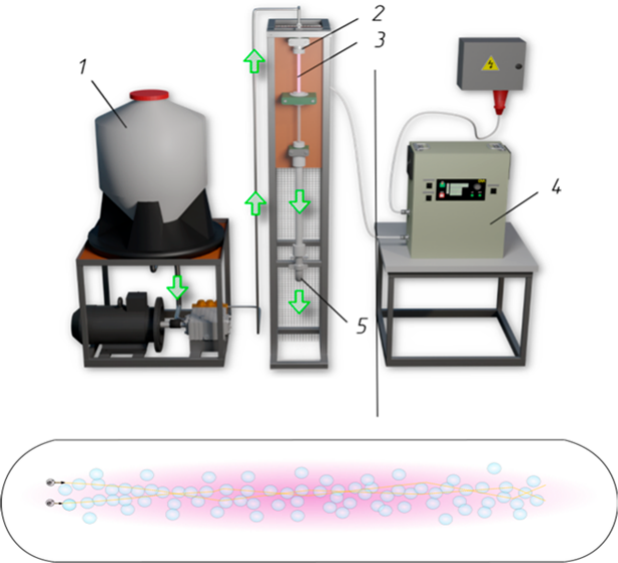

A theoretical model for an electrical discharge in a cavitating liquid is developed and compared with experiments for the optimization of the water treatment device. The calculations based on solution of the Noltingk—Neppiras equation support the hypothesis that the electric field promotes the formation of vapor microchannels inside a liquid gap between the electrodes, where at a low gas pressure Paschen’s conditions of rupture and abnormal glow discharge maintenance in those microchannels are fulfilled. Theoretical analysis of the cavitation processes and the discharge formation processes is in qualitative agreement with the experimental data obtained in this work in a water treatment device using a hydrodynamic emitter. The following graphic illustrates the experimental setup: (1) feeding tank, (2) hydrodynamic emitter, (3) zone of cavitation inside the plasma reactor, (4) high-frequency generator of electric impulses, and (5) outlet.

New technologies for contaminated-water treatment are currently in great demand. The proposed approaches include oxidation-based physical and chemical processes,^1−13^ in particular methods that involve the simultaneous action of UV radiation and oxidizers: ozone,^14−18^ hydrogen peroxide,^25^ etc., and those that involve various combinations of ozonation and sonication.^19−27^

Among such technologies for purification of aqueous solutions from organic compounds methods that involve the use of combined plasma-catalytic processes are especially interesting.^28^ Experiments have shown that in a liquid subjected to an intense ultrasound (US) field above the cavitation threshold, there may exist a form of electric discharge that features volumetric glow across the entire space between the electrodes.^29^ Such discharge can be used to treat liquid flows in order to remove organic contaminants and microorganisms from contaminated water,^30,31^ for carbon dioxide conversion,^32^ etc. Such a method does not require additional use of reagents.

When processing the liquid flow by the above-mentioned cold plasma method, local electrical breakdowns occur in the liquid flow with the formation of streamers containing an ionized oxygen and hydrogen mixture. Therefore, in order to develop an effective method of wastewater treatment, it is necessary to create a favorable environment for discharge to occur simultaneously throughout the entire volume of the flow reactor. In order to optimize the characteristics of the process, i.e., the optimal intensity of ultrasound in the discharge zone, it is necessary to understand the processes that occur in such a hybrid environment.

Recently, the phenomena of the plasma discharge in liquids at intensive ultrasonic field above the cavitation threshold attracted great interest.^33−41^ At the same time, a theoretical model for such processes is lacking. The main purpose of this work is to formulate such a model and test it using experimental data from a water treatment device with a hydrodynamic emitter.

In ref (29) we have formulated the hypothesis that such discharge can emerge in a cavitating liquid because it is a dynamic two-phase medium. In that case, the following scenario for the development of such a discharge may be suggested: in a medium with developed cavitation, numerous unstable oscillating bubbles may occur. The electric field promotes the arrangement of such bubbles into chains, with the formation of numerous gas microchannels in the gap between the electrodes, where at a low gas pressure Paschen’s conditions of rupture and abnormal glow discharge maintenance in those microchannels are fulfilled. Those microchannels are dynamic formations that constantly appear and disappear in the US acoustic and quasi-stationary or stationary electric field, creating the image of average volumetric discharge glow.

In this work, we introduce detailed theoretical analysis of the cavitation processes and the discharge formation processes, which enables us to optimize the water treatment device for removal of organic contaminants from water flows.

First of all, let us consider the constrained medium (space between the electrodes) filled with a liquid (water). If we apply an external sound field to the medium, we expect to observe the creation and subsequent oscillations of the bubbles. This happens due to the expansion and contraction phases of the cavities in the liquid under an external periodic sound field. Assuming the created cavities are filled with gas (generally air), we can expect two types of cavitation dynamics of the bubbles: stable and transient. More detailed studies of distinctions between these two types of cavitation can be found in ref (42). Transient cavitation is characterized by the eventual collapse of the bubble. Importantly, during the transient cavitation, the bubble’s size can grow orders of magnitude before collapsing.

The basic features of the cavitating bubbles can be described by the Noltingk—Neppiras equation^42^
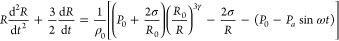
1where *P*_0_ is the initial pressure in the cavity [Pa] (10^5^ Pa), *P*_A_ is the acoustic pressure amplitude [Pa], ω is the frequency of acoustic radiation [2π Hz], *R*_0_ is the initial radius of the cavity [m], ρ_0_ is the density of the liquid [kg/m^3^] (997 kg/m^3^ for water), and σ is the surface tension [N/m] (72.86 × 10^–3^ N/m for water at 20 °C).

Assuming an ideal gas in the bubbles, and neglecting heat and mass transfer, we can write the expression for the pressure inside the cavity as
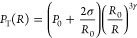
2After solving the nonlinear eq 1, we find that for the operating regime with ultrasound frequencies of the order of 20–60 kHz and the sound pressure amplitude of 0.7 MPa (which corresponds to the ultrasound intensity of approximately 14 W/cm^2^), for various initial bubble radii *R*_0_, an increase in the radius to values from *R*/*R*_0_ ∼ 5 to *R*/*R*_0_ ∼ 100 is observed. Figure 1a shows the bubble growth curves on a logarithmic scale. It can also be seen that at a certain moment the cavity collapses, reflecting the transient cavitation type of the bubbles. Knowing that the size of the cavitating bubbles can grow in orders of magnitude, depending on initial radius *R*_0_ we can estimate the pressure inside the bubble as *P*_T_/*P*_0_ ≈ (*R*/*R*_0_)^−3^, which gives the pressure drop up to *P*_T_/*P*_0_ ∼ 10^–6^. Figure 1b shows the dynamics of the bubble size with *R*_0_ = 10 μm for different acoustic field period *T* = 2π/ω. Under the assumption that the concentration of bubbles is sufficient for the formation of plasma flow channels, we may expect that comparable pressure is maintained in the bubbles forming the plasma channels. Keeping this in mind, we turn to Pachen’s law for the voltage breakdown in gases^39^
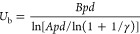
3where *A* and *B* are the coefficients determined from the experiment, *p* is the gas pressure, *d* is the distance between electrodes, and γ is the coefficient of secondary emission—the number of electrons leaving the cathode per incident positive ion. Now we compare the experimental data for the breakdown voltage in the region between two electrodes with the theoretical Pachen’s curves (Figure 2). Here we assume that the electric field in water is smaller by a factor of ε_r_ ≈ 74; thus, we divide the result of eq 3 by ε_r_ and plot the corresponding theoretical plot in Figure 2.

**Figure 1:**
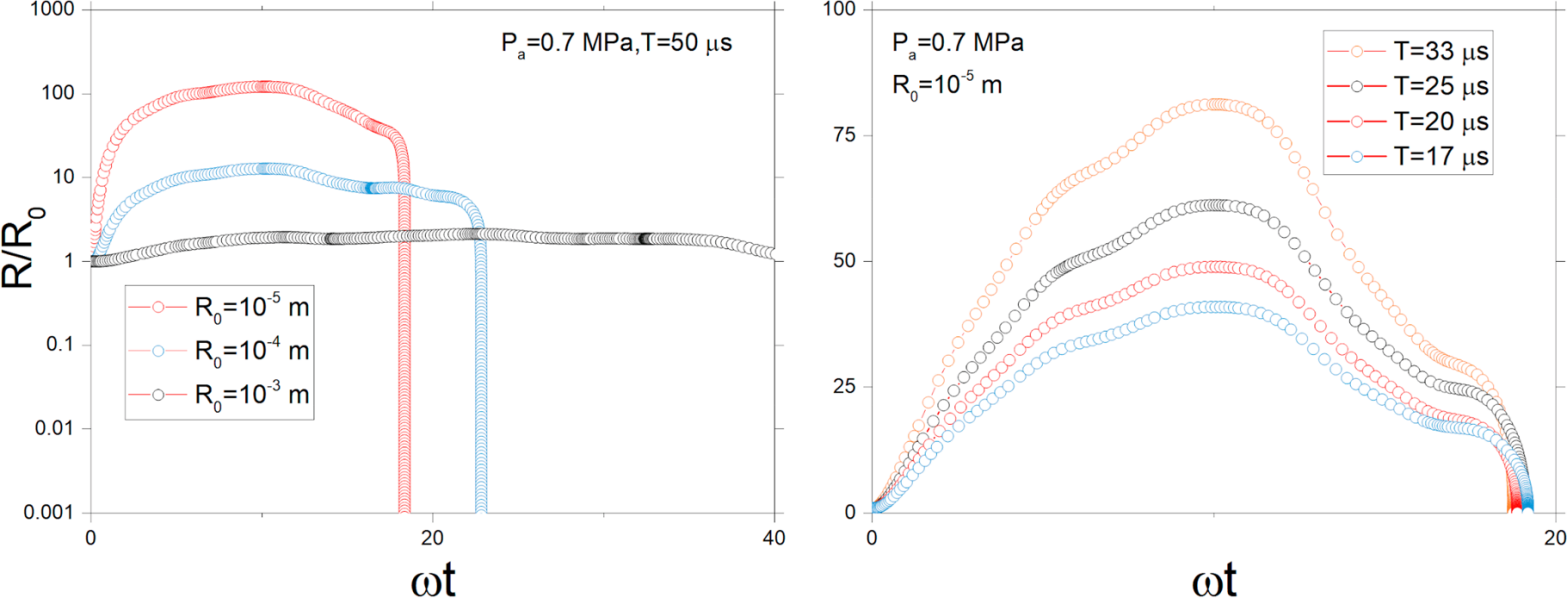
(a) Simulated dynamics of a single bubble of radius *R* normalized by the initial radius *R*_0_ for three different values of *R*_0_. Other parameters of the calculations are *P*_a_ = 0.7 MPa, *T* = 2π/ω = 50 μs. (b) Simulated dynamics of a bubble with initial radius *R*_0_ = 10^–5^m calculated for various periods *T*.

**Figure 2:**
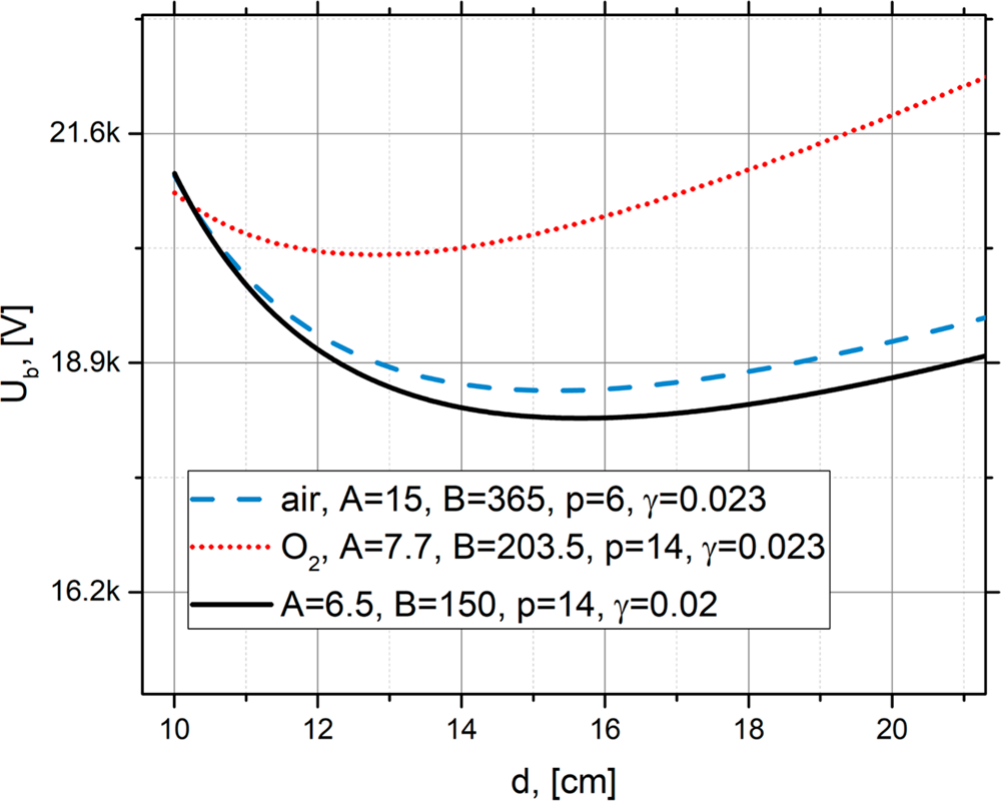
Pachen’s curve calculated for three sets of parameters *A*, *B*, γ, and *p*. *A* and *B* have the same dimensions [cm Torr]^−1^; *p* is measured in [Pa], and γ is dimensionless. The parameters *A* and *B* which correspond to blue dashed line typically used for air environment and those for black solid line are taken between the typical parameters for oxygen and hydrogen.^39^

Based on the theoretical model described above, we can conclude that in order to treat the whole liquid flow with the maximum intensity at the lowest energy consumption, we need to create with the help of ultrasonic cavitation a uniform cloud of pulsating bubbles, which fills the entire space between the electrodes. To form such a cavitation region in the fluid flow, we chose to use hydrodynamic emitters. Such emitters have a number of technological advantages such as ease of manufacture, high reliability (due to the absence of electronic components), and low cost of acoustic energy. At the same time, acoustic effects, such as cavitation and pulsation, arising during the operation of a hydrodynamic emitter, are the main factors determining the possibility of plasma formation. To optimize the length of the reactor working area, a long quartz tube was used in which the dimensions of the cloud of cavitation bubbles formed during the operation of the hydrodynamic emitter were visually determined. Experimentally, it was found that the dependence of the size of the cavitation region on the pressure of the liquid supplied to the hydrodynamic radiator is essentially nonlinear. At low supply pressures, a smooth increase in its length was observed; then at pressures reaching about 5–6 MPa, the bubble torch length increased by 3–4 times with a sharp jump because the hydrodynamic radiator apparently went into a different mode of operation.

The hydrodynamic emitter caused a hydrostatic pressure drop from 3 to 6 MPa to 0.07–0.02 MPa, which caused vibrations in a wide frequency range from 0.3 to 60 kHz with an intensity of 2.5–15.5 W/cm^2^ (which corresponds to acoustic pressure levels of 0.29–0.7 MPa). Thus, the parameters of the medium match the parameters, which were taken for the modeling of the process above.

After the approximate length of the cavitation cloud was determined, electrodes were placed at the ends of the tube so that one of the contacts was at the emitter while the other contact was on the opposite side of the reactor. The reactor length of 150 mm was chosen because the maximum length of the cavitation zone for the used hydrodynamic emitter was found to be 150 mm. As a result, plasma could be formed in the experimental setup along the entire length of the reactor. The minimum pressure at which the plasma was ignited was 3 MPa, and the full length of the torch was observed at only 6 MPa. At inlet pressures below 6 MPa, which corresponds to hydrostatic pressures near the emitter outlet above 0.02 MPa, the plasma discharge existed only in a part of the reactor because the bubbles necessary for the process did not reach the electrode at the other end.

We equipped our experimental setup with the following measuring devices: (i) The pressure at the outlet of the hydrodynamic emitter was measured with a FIZTEKh VTIf vacuum gauge (accuracy class 0.6 in the measurement range from 0 to 100 kPa). (ii) The voltage was measured using a Tektronix TPS2024 oscilloscope. (iii) The plasma glow intensity was measured with an Ocean Optics QE65000 optical spectrometer in the wavelength range from 220 to 1100 nm. (iv) Acoustic pressure data were taken with dynamic pressure sensor GTLab 5 V110TB-6. During the experimental runs, it was noted that stable discharge was possible only when an alternating voltage with a frequency higher than 25 kHz was used for the power supply. This could indicate that the lifetime of a bubble channel does not exceed 40 μs, which is in good agreement with the presented model.

Studies were performed to monitor the changes in the current–voltage characteristics during ignition and stable discharge. For this purpose, an alternating voltage with a frequency of 38 kHz was applied to the electrodes. The amplitude of the voltage was increased step by step. We used an oscilloscope to monitor the discharge characteristics. The obtained oscillograms are shown in Figure 3. It can be seen that when a breakdown occurs, voltage decreases abruptly, and at the same time the current increases. Oscillograms taken during the stable phase of the sonoplasma discharge also clearly show that the voltage values reach their peak values and then fall (Figure 3b).

**Figure 3:**
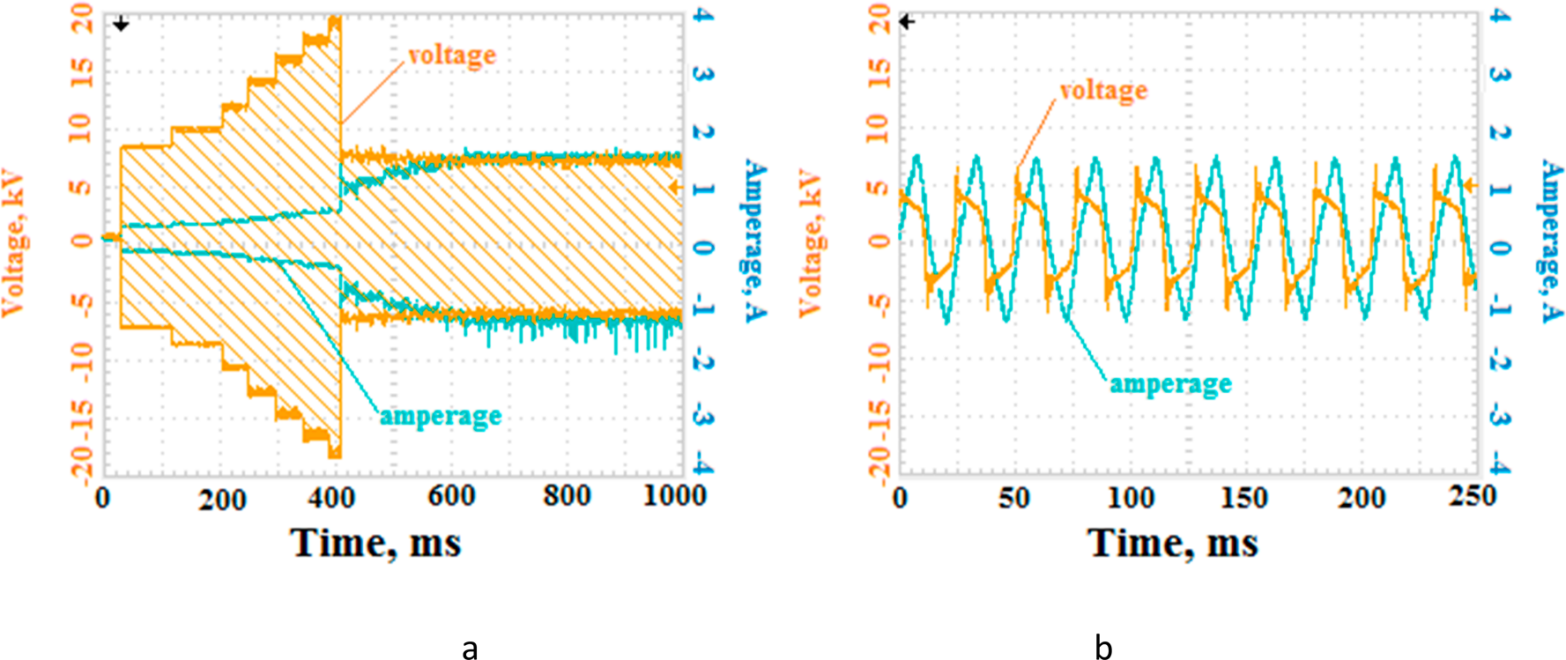
Changes in the values on the voltage and current waveforms at the time of ignition of the plasma discharge (a) and the voltage and current waveforms during the stable phase of the plasma discharge in a reactor 150 mm long.

We have found that both the breakdown voltage and the stable discharge voltage decreased with decreasing pressure at the outlet to the hydrodynamic emitter, as shown in Figure 4. Lower hydrostatic pressure at the outlet corresponds to a higher pressure drop in the emitter, i.e., a higher acoustic pressure, which reaches the target acoustic pressure of 0.7 MPa at optimal conditions (pressures at the outlet below 0.02 MPa and above 6 MPa at the inlet of the emitter).

**Figure 4:**
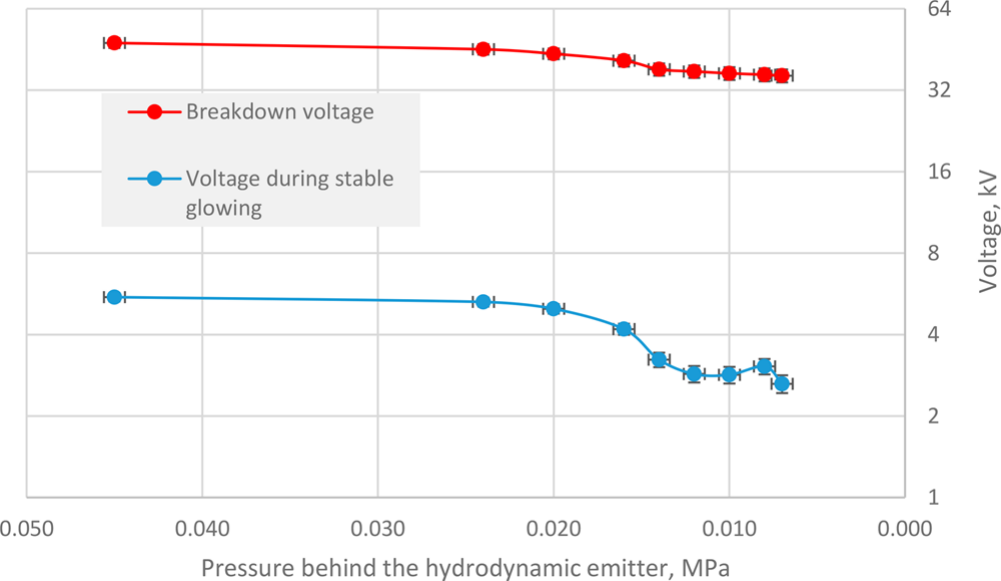
Dependence of breakdown voltage (red curve) and voltage during stable discharge glow (blue curve) on the hydrostatic pressure at the outlet to the hydrodynamic emitter.

Important information about the plasma discharge process can be obtained by examining the radiation spectra. A typical spectrum of the sonoplasma discharge is shown in Figure 5.

**Figure 5:**
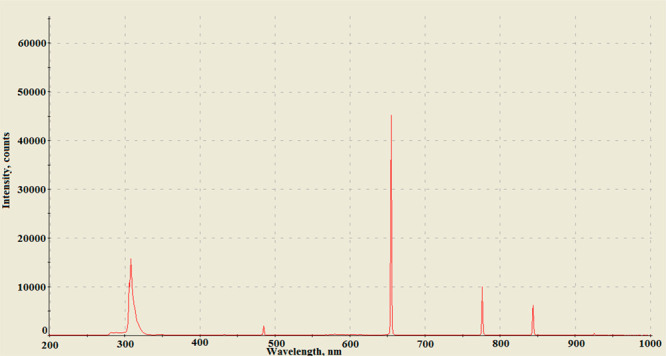
Radiation spectrum of a plasma discharge formed in water in the field of hydrodynamic cavitation.

The effect of the pressure conditions on the intensity of the plasma glow was investigated. The experiment was performed in the inlet pressure range of 4.0–7.5 MPa, which corresponds to pressures at the outlet of 0.045–0.015 MPa, i.e., a pressure drop of 3.955–7.485 MPa. The results of the experiment are shown in Figure 6.

**Figure 6:**
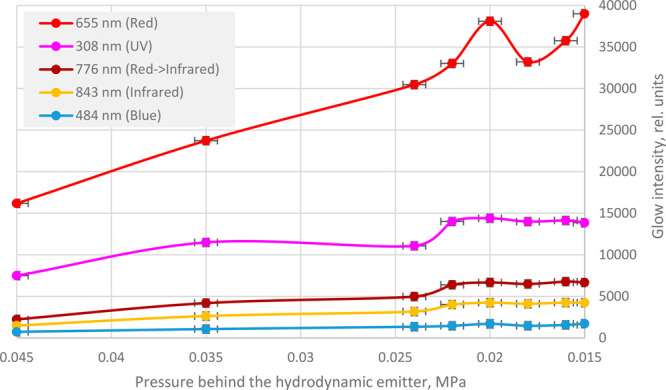
Dependences of the radiation intensity on the pressure at the outlet of the hydrodynamic emitter.

As shown in Figure 6, the intensity of the glow in the ultraviolet, visible, and infrared ranges does not further increase after reaching its optimal value at a pressure of 0.02 MPa at the outlet of the emitter.

The comparison of the dependence of the breakdown voltage on the distance between the electrodes with the theoretical model developed in this work is shown in Figure 7. The theoretical fit gives us approximate values of the gas pressure inside the formed streams between *P* = 6 Pa and *P* = 14 Pa, which we may assume as the pressure inside the bubbles. These estimated pressures are in a qualitative agreement with the pressures obtained by solving eq 1 for the considered range of parameters; i.e., the pressure of the order of several Pa in the cavitating bubbles is predicted for initial radii of the order between *R*_0_ ∼ 10^–4^ m and *R*_0_ ∼ 10^–5^ m (see Figure 1). Thus, the estimated characteristics support the qualitative picture of plasma discharge in the cavitating medium.^44^

**Figure 7:**
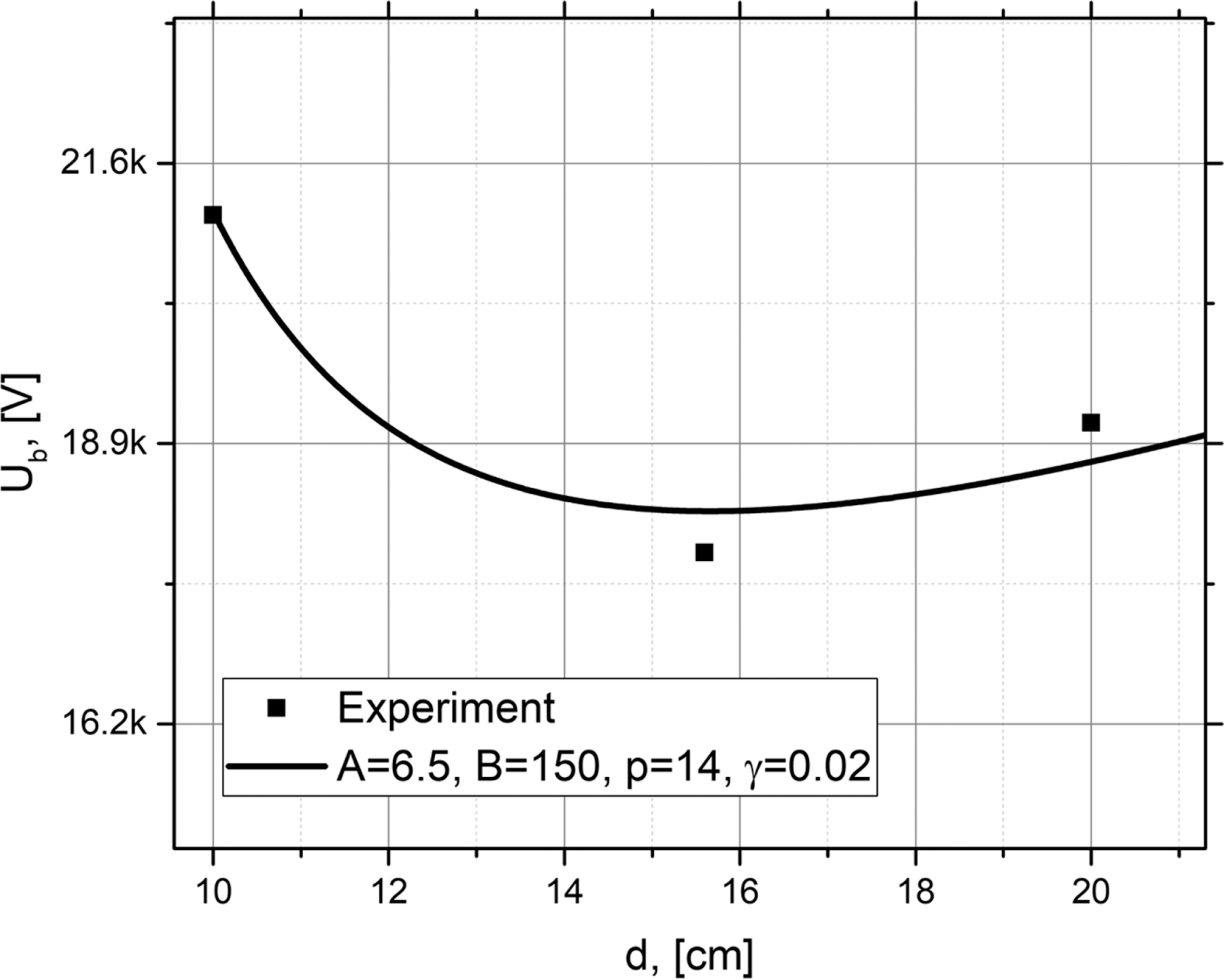
Comparison of theory (solid curve) with experimental data of the breakdown voltage between the electrodes (black squares).

From Figure 7 we can conclude that for the given range of pressures the parameters which are between the oxygen and hydrogen environment give quite good agreement with the experiment. This may be interpreted by the presence of the mixed gas environment inside the cavitating region.

Based on the theoretical analysis of the cavitation processes inside a reactor for water treatment and the discharge formation processes between two electrodes placed at the ends of a cavitation zone, we were able to optimize a water treatment device for the removal of organic contaminants from water flows. The device contained a reactor with a hydrodynamic emitter to cause cavitation inside the water flow. Two electrodes were placed at the ends of the reactor to cause plasma discharge inside the channels formed by the cavitation bubbles.

Theoretical and experimental analyses enabled us to optimize the following parameters: (i) reactor length of 150 mm, which corresponded to the maximal length of the bubble torch; (ii) minimal voltage frequency of 25 kHz, which corresponded to the lifetime of the bubble channels of 40 μs; (iii) optimal pressure drop at the hydrodynamic emitter of 5.98 MPa, which corresponded to an acoustic pressure of 0.7 MPa in the cavitation zone. The determined parameters can be used to minimize the energy consumption of a water treatment device. Moreover, the obtained results can make it possible to implement the elaborate process of water purification on a large scale.
